# Predictors of under-five healthcare utilization in Rongo sub-county of Migori County, Kenya: results of a population-based cross-sectional survey

**DOI:** 10.11604/pamj.2022.41.108.31618

**Published:** 2022-02-08

**Authors:** Ash Lauren Rogers, Aaron Xian Ti Lee, Jamie Gudeon Joseph, Joseph Robert Starnes, Tom Otieno Odhong, Vincent Okoth, Julius Mbeya, Troy Moon

**Affiliations:** 1 Lwala Community Alliance, Rongo, Migori County, Kenya,; 2 Department of Biostatistics, Vanderbilt University, Nashville, Tennessee, United States of America,; 3 Vanderbilt Institute for Global Health, Vanderbilt University Medical Center, Nashville, Tennessee, United States of America; 4 Department of Health Services, Migori County, Kenya

**Keywords:** Health care utilization, community health workers, child health, Kenya

## Abstract

**Introduction:**

to achieve the sustainable development goal for child survival, we must better understand the socioeconomic characteristics, household behaviors and access to community health services which predict care utilization for children. This study assessed predictors of health care utilization for children under five in Migori County, Kenya.

**Methods:**

we used multivariable logistic regression in the context of an integrated health intervention which employed paid, trained, and supervised community health workers (CHWs), inclusive of traditional birth attendants (TBAs). The intervention was delivered with Ministry of Health in one of five geographies included in the study.

**Results:**

community health workers (CHW) home visits were associated with a two-fold increase in care seeking for children with respiratory symptoms. Following implementation of a CHW-led malaria intervention, the use of malaria rapid diagnostic tests increased, while fever prevalence decreased. Households in the intervention area were three times more likely to seek care for their child´s fever. Increased care utilization for children with fever was positively associated with male partner attendance at antenatal care visits and negatively associated with skilled delivery and recognition of warning signs. Care utilization for respiratory symptoms was positively associated with caregiver education and negatively associated with household size. Care utilization for diarrhea was positively associated with having a recent under-five death in the household.

**Conclusion:**

the study suggests that trained and motivated CHWs may be an effective tool for improving care utilization for children. Further, the study builds on evidence of male partner involvement and caregiver education as predictors of child care utilization.

## Introduction

Globally, under-five mortality has reduced from 93 deaths per 1,000 live births in 1990 to 38 in 2019 [1]. While this represents progress, gains have not been equally distributed, with wide disparities both between regions and countries, as well as within countries. In Kenya, for example, reductions in under 5 (U5) mortality vary significantly by county, with the national rate in 2014 being 51 deaths per 1,000 live births and rates by county ranging from 20 to 100 deaths per 1,000 live births [2-4]. The Sustainable Development Goals (SDGs) set targets for further reducing U5 mortality to less than 25 deaths per 1,000 live births and ensuring universal health coverage (UHC) by 2030 [5]. Meeting these goals depends on better understanding the factors that limit health access and utilization at regional and local levels.

The majority of childhood deaths in Kenya are caused by infectious, preventable, and treatable conditions including malaria, acute respiratory infection (ARI), and diarrhea [6]. All of these illnesses have well-established and affordable methods for treatment and/or prevention. However, these services are not reaching all children, especially in rural and hard to reach areas. There are large disparities in health utilization between regions in Kenya. According to the 2014 Kenya Demographic Health Survey (KDHS), U5 care utilization ranged from 35% to 71% for ARI, 50% to 75% for fever, and 44% to 65% for diarrhea. Barriers to health access likely contribute to these disparities. Roughly 54% of Kenyans in rural areas report experiencing at least one problem accessing care, compared to a rate of 34% amongst their counterparts in urban areas. In Nyanza province, this rate is 60%, compared to 46% across the country as a whole [4].

Universal health coverage (UHC) is one of the pillars of the “Big Four Agenda” declared by the president of Kenya in a promise to push major reforms in 2017 [7]. This agenda centers primary healthcare as an approach to achieving UHC. However, primary health services in Kenya face significant challenges including health workforce shortages, distance to care, lack of trust between communities and the formal health system, and reliance on traditional healers and traditional birth attendants, to name a few. Community health systems and community health workers (CHWs) are intended to bridge these gaps but have suffered from fragmentation and underinvestment. Thus, access to a CHW varies greatly across the country. Kenya´s Community Health Strategy 2020-2025 sets a path for ambitious improvements in community health and greater alignment with World Health Organization (WHO) guidelines, namely greater coverage, compensation, training, supervision, and equipping of CHWs [8].

This study takes place in the context of an integrated community health intervention which employed professionalized CHWs, inclusive of traditional birth attendants (TBAs). The intervention mapped all practicing TBAs in the region and incorporated them within existing cadres of government CHWs. Together, they were paid, trained, supervised, equipped and connected to local health facilities in line with WHO guidelines and the CHW Assessment and Improvement Matrix (AIM) tool [8-10]. The intervention was implemented by Lwala Community Alliance (Lwala) in partnership with the Migori County Ministry of Health. We aim to explore how socioeconomic characteristics, household (HH) behaviors and access to community health services predicts U5 care utilization for fever, diarrhea and ARI symptoms in rural Migori County. These are critical insights to inform local and regional planning, policy and interventions aimed at advancing UHC and reducing U5 morality.

## Methods

**Study design:** we used cross-sectional data from two population-based surveys collected from HHs within six sub-locations of Migori County, located in Western Kenya on the border with Lake Victoria to the west and Tanzania to the south. These surveys were conducted for the purpose of determining the status of health and socioeconomics metrics of this population. The full protocol has been presented elsewhere [11]. Data collection was first carried out in the North Kamagambo (NK) ward of Rongo sub-county in 2017. A subsequent survey with an expanded geographic region covering all four wards of Rongo sub-county was then carried out in NK and East Kamagambo (EK) in May 2018, followed by Central Kamagambo (CK) and South Kamagambo (SK) in January and February 2019. Finally, two wards, West and Central Kanyamkago, in Uriri sub-county, were included as control locations and were surveyed in June and July 2019. Information on demographics, CHW visitation and health seeking behaviors for HHs with U5 children reporting fever, diarrhea, or respiratory illness in the two weeks prior to being surveyed were specifically extracted for analysis for this study. Data presented below are predominantly from the survey conducted in 2018/2019, with data from 2017 presented to show comparisons across time in NK in terms of healthcare utilization for fever, diarrhea, and ARI (Annex 1, Annex 2, Annex 3).

Details of the sampling strategy and household selection are presented elsewhere [11-15]. Briefly, a modified version of the World Health Organization Expanded Programme of Immunization method was used [16,17]. Each ward was divided into the appropriate number of squares to achieve the calculated sample size. Within each square, enumerator teams used the spin-the-bottle technique to determine household selection [16]. This minimized the biases associated with the traditional spin-the-bottle method [18] by using the center of an arbitrary square rather than the center of a town. Male heads of HH were interviewed only if it was reported that no female head of HH was available. If no head of household was present or the head of household was < 18 years old, the interviewer skipped this house going to the next HH along the line selected.

**Survey tool:** the survey contained more than 300 questions across multiple domains and was modeled on validated tools, including the KDHS (Annex 1, Annex 2, Annex 3) [4]. The tool was administered to all respondents, utilizing branching logic based on the respondent's gender, age, and children in the HH. Survey responses were de-identified, and participants received 50 KES (~$0.50 USD) as incentive in cellphone airtime for their time. Trained interviewers administered the surveys. All were hired from the community and were fluent in English, Dholuo, and Swahili. Surveys were administered on tablets using a Research Electronic Data Capture (REDCap) hosted at Lwala to reduce data entry errors and ensure appropriate skip logic [19,20].

**Statistical analysis:** data analysis was performed in R version 3.6.1 and R Studio version 1.2.1335. Descriptive statistics were calculated for categorical variables as unweighted percentages. Logistic regression using the maximum likelihood approach was employed. Three separate models were fit, corresponding to the three outcomes of interest: seeking medical advice for fever, for diarrhea, and for ARI. This method assumes that the data follow a binomial probability distribution, and it defines a likelihood function that determines the probability of the outcome of interest given the model and the data. A model-based standard error estimator assumes that the mean-variance relationship is correct, and the model allows for the possibility of a mis-specified mean model. Furthermore, because households were sampled randomly and interviewed only once, all observations and errors are assumed to be independent. It also is assumed that all covariates are independent and that there is no multicollinearity among the variables. Odds ratios (OR) with 95% confidence intervals (CI) were reported. The significance level for all tests was two-sided and set at 0.05.

**Ethical considerations:** ethics review and approval were received from the AMREF Ethics and Scientific Review Committee (P452/2018) and the Vanderbilt Institutional Review Board (#161396).

## Results

**Demographics:** a total of 4,137 HHs with at least one child U5 were included in this analysis (Table 1). The median age of respondents was 29 years, and respondents were predominantly female (83%). A majority reported being married monogamously (78%), with a smaller number married polygamously (11%). Over half reported not having education beyond primary school (56%). There was a notable regional difference in education, with higher rates of education in CK. Seventh Day Adventist was the predominant religion (39%), followed by Protestant (22%), the African-initiated Christian religion called Roho Church (16%), and Catholic (15%). There were regional differences by religion, with NK having more Roho Church adherents and CK having more Seventh Day Adventists. The median HH size was 4.8 persons, with the majority of HH incomes coming from self-employment (60%) and agriculture (23%). Most HHs reported that their children slept under a bed net the night prior to being interviewed (92%). Further, most HHs used firewood or charcoal as a fuel source (91%). While many had a separate ventilated kitchen (41%), others had less advantageous cooking locations including: separate kitchens without ventilation (16%), in-home with ventilation (13%), and in-home without ventilation (9%).

**Table 1:** descriptive statistics for the heads of households in which at least one child under-five years old lived: 2018/2019

Variable N (%)	NK N=197	EK N=619	CK N=1133	SK N=1115	Uriri N=1073	Total N=4137
Age median [IQR|	28 [24,32]	29 [24,35]	29 [24,34]	30 [25,35]	29 [24,33]	29 [24,34]
**Gender**						
Male	42 (21)	167 (27)	149 (13)	181 (16)	174 (16)	713 (17)
Female	155 (79)	451 (72)	981 (86)	934 (84)	899 (84)	3420 (83)
Missing	0 (0)	1 (<1)	3 (<1)	0 (0)	0 (0)	4 (<1)
**Marital status**						
Never married	4 (2)	17 (3)	58 (5)	48 (4)	26 (2)	153 (4)
Married monogamous	157 (79)	475 (77)	905 (80)	882 (80)	825 (77)	3244 (78)
Married polygamous	28 (14)	108 (17)	88 (7)	89 (8)	146 (14)	459 (11)
Cohabitating	1 (<1)	0 (0)	9 (<1)	12 (1)	4 (<1)	26 (<1)
Separated	1 (<1)	1 (<1)	17 (1)	11 (1)	7 (<1)	37 (<1)
Divorced	0 (0)	2 (<1)	7 (<1)	0 (0)	5 (<1)	14 (<1)
Widowed	6 (3)	15 (2)	49 (4)	73 (6)	60 (6)	203 (5)
Missing	0 (0)	1 (<1)	0 (0)	0 (0)	0 (0)	1 (<1)
**Highest education level**						
No education	3 (1)	6 (<1)	5 (<1)	7 (<1)	9 (<1)	30 (<1)
Class 1 -8	136 (69)	410 (66)	472 (42)	598 (54)	695 (65)	2311 (56)
Form 1-4	50 (25)	155 (25)	459 (40)	406 (37)	306 (29)	1376 (33)
Some college - post-graduate	7 (4)	47 (8)	197 (17)	104 (9)	63 (6)	418 (10)
Missing	1 (<1)	1 (<1)	0 (0)	0 (0)	0 (0)	2 (<1)
**Religion**						
No religion	0 (0)	1 (<1)	3 (<1)	2 (<1)	1 (<1)	7 (<1)
Catholic	33 (17)	107 (17)	135 (12)	123 (11)	243 (22)	641 (15)
Seventh Day Adventist	58 (29)	239 (38)	603 (53)	501 (45)	221 (20)	1622 (39)
Protestant	19 (9)	130 (21)	186 (16)	272 (24)	332 (31)	939 (22)
Roho Church	76 (38)	106 (17)	139 (12)	149 (13)	202 (18)	672 (16)
Legio Maria	5 (3)	11 (1)	17 (1)	3 (<1)	20 (1)	56 (1)
African Independent Church	5 (3)	21 (3)	36 (3)	50 (4)	50 (4)	161 (4)
Other	1 (<1)	4 (<1)	13 (1)	14 (1)	1 (<1)	33 (<1)
Missing	0 (0)	1 (<1)	1 (<1)	1 (<1)	3 (<1)	6 (<1)
Household size, median persons	5.0	5.2	4.7	4.8	4.6	4.8
**Main source of household income**						
Self-employed	120 (61)	373 (60)	740 (65)	719 (64)	536 (50)	2488 (60)
Employment by other	37 (18)	109 (17)	258 (23)	127 (11)	135 (12)	666 (16)
Farming/livestock	39 (19)	128 (21)	110 (10)	261 (23)	393 (37)	931 (23)
Other	1 (<1)	9 (1)	18 (1)	7 (<1)	3 (<1)	38 (<1)
Missing	0 (0)	0 (0)	7 (<1)	1 (<1)	6 (<1)	14 (<1)
**Fuel used for cooking**						
Firewood/charcoal	188 (95)	589 (95)	922 (81)	1023 (92)	1042 (98)	3764 (91)
Parafin/gas/electric/other	9 (5)	27 (4)	210 (19)	87 (7)	19 (1)	352 (9)
Missing	0 (0)	3 (<1)	1 (<1)	5 (<1)	12 (1)	21 (<1)
**Location of cooking**						
Outdoors	50 (25)	139 (22)	167 (14)	208 (18)	255 (24)	819 (20)
Separate kitchen-ventilated	89 (45)	292 (47)	388 (34)	487 (43)	440 (41)	1696 (41)
Separate kitchen-not ventilated	18 (9)	55 (9)	161 (14)	242 (22)	199 (18)	675 (16)
In home-ventilated	24 (12)	87 (14)	216 (19)	102 (9)	121 (11)	550 (13)
In home-not ventilated	15 (8)	39 (6)	194 (17)	71 (6)	45 (4)	364 (9)
Other	0 (0)	1 (<1)	3 (<1)	0 (0)	0 (0)	4 (<1)
Missing	1 (<1)	6 (<1)	4 (<1)	5 (<1)	13 (1)	29 (<1)
Child slept under bed net previous night	189 (95)	577 (93)	1069 (94)	1025 (92)	957 (89)	3817 (92)

**Fever:** we looked at common health related issues for U5 children (fever, diarrhea, and ARI) and questioned the head of HH on health seeking behaviors related to each condition (Table 2). Across the areas surveyed, respondents were first questioned if their child had experienced a fever in the two weeks prior to being surveyed, with NK and EK each reporting “yes” in slightly more than 25%, followed by CK (20%), Uriri (15%) and SK (13%) respectively. In NK and EK, as well as Uriri, more than 70% of those that reported their child had a fever stated they sought care for the fever at a health facility. Finally, of those seeking care for a fever, more than 80% from NK and Uriri reported having had a malaria rapid diagnostic test (RDT) performed, compared to roughly 65% of those from EK, CK and SK.

**Table 2:** healthcare utilization for households with children under-five years of age: 2018/2019

Variable N (%)	NK N=197	EK N=619	CK N=1133	SK N=1115	Uriri N=1073	Total N=4137
Child has had fever in last 2-weeks	51 (26)	169 (27)	229 (20)	147 (13)	163 (15)	759 (18)
Sought advice or treatment for fever	40 (78)	118 (70)	135 (59)	95 (64)	121 (74)	509 (67)
Malaria rapid diagnostic test (RDT) was performed	35 (88)	81 (67)	91 (67)	62 (65)	99 (82)	368 (72)
Child has had diarrhea in last 2-weeks	18 (9)	87 (14)	98 (9)	67 (6)	51 (5)	321 (8)
Sought advice or treatment for diarrhea	9 (50)	44 (51)	53 (54)	33 (49)	33 (65)	172 (54)
Child has had breathing problem* in last 2-weeks	48 (24)	145 (23)	172 (15)	114 (10)	98 (9)	577 (14)
Sought advice or treatment for breathing problem	31 (65)	67 (46)	86 (50)	62 (54)	65 (66)	311 (54)
Ever visited by a community health worker (CHW), identifies signs child needing medical attention	126 (64)	180 (29)	295 (26)	438 (39)	345 (32)	1348 (33)
No correct responses	2 (1)	13 (2)	23 (2)	41 (4)	36 (3)	115 (3)
One to two correct responses	80 (41)	246 (40)	302 (27)	233 (21)	238 (22)	1099 (26)
Three or more correct responses	115 (58)	360 (58)	808 (71)	841 (75)	799 (75)	2923 (71)

*Breathing problem: cough or difficult or shallow breathing

We also compared the NK data from 2018/2019 to the survey data collected in 2017 (Table 3). The proportion of children reporting fever in the two-weeks prior to being surveyed dropped from 50% in 2017 to 26% in 2018/2019. Amongst respondents who reported fever, care seeking was relatively unchanged from 2017 (82%) to 2018/2019 (78%). However, of those seeking care for fever, 88% reported having a malaria RDT performed in 2018/2019, compared to only 24% in 2017. The proportion of children reporting diarrhea in the two-weeks prior to being surveyed dropped from 26% in 2017 to 9% in 2018/2019, while the proportion of children reporting an ARI dropped from 40% in 2017 to 24% in 2018/2019. Despite drops in the overall proportion of cases of either diarrhea or ARI between survey time points, those who then reported seeking care for these conditions remained roughly the same at 50-65%.

**Table 3:** healthcare utilization for households with children under-five years of age in North Kamagambo

Variable N (%)	2017 N=272	2018/2019 N=197
Child has had fever in last 2-weeks	136 (50)	51 (26)
Sought advice or treatment for fever	112 (82)	40 (78)
Malaria rapid diagnostic test (RDT) was performed	27 (24)	35 (88)
Child has had diarrhea in last 2-weeks	72 (26)	18 (9)
Sought advice or treatment for diarrhea	42 (58)	9 (50)
Child has had breathing problem* in last 2-weeks	109 (40)	48 (24)
Sought advice or treatment for breathing problem	60 (55)	31 (65)
Ever visited by a community health worker (CHW), identifies signs child needing medical attention	221 (81)	126 (64)
No correct responses	3 (1)	2 (1)
One to two correct responses	99 (36)	80 (41)
Three or more correct responses	170 (63)	115 (58)

* Breathing problem: cough or difficult or shallow breathing

**Diarrhea:** in the two weeks prior to being surveyed in 2018/2019, diarrhea prevalence overall was 8% across the regions sampled, ranging from a low of 5% in Uriri, to as high as 14% in EK. Overall, roughly 50% of HHs reported seeking care for their child´s diarrhea, with only Uriri having a slightly higher proportion of care seeking at 65%.

**Acute respiratory infection:** the prevalence of ARI in 2018/2019, defined as having had cough and/or difficulty breathing in the two weeks prior to survey collection, was 14% for all areas surveyed, with NK and EK reporting >20% and Uriri, CK and SK reporting between 9-15%. In both NK and Uriri, care seeking for ARI was reported in approximately 65% of cases, while in all remaining areas care seeking for an ARI averaged only around 50%.

**CHW visits:** in addition to questions related to care seeking for the above conditions, we also questioned the head of HH about whether their HH had ever received a visit from a CHW. In NK, 64% of respondents reported their HH had ever been visited by a CHW in 2018/2019, compared to only 26-39% for all other areas surveyed.

**Identification of warning signs:** finally, we asked respondents to identify different scenarios which could indicate whether a child needs to be seen by a health professional or not. Across geographic areas, roughly 97% of all respondents could identify at least one to two conditions suggestive of when to seek medical attention. Further, approximately 70% of respondents in SK, CK, and Uriri could identify three or more conditions suggestive of need to seek medical attention, while only 58% of respondents from NK and EK could identify three or more conditions.

**Predictors of care utilization:** in multivariable logistic regression models, the characteristics most associated with health-care utilization varied by illness (Table 4). For children with fever, a HH from NK had a three-fold higher likelihood of seeking care for the fever (OR = 3.27; 95% CI = 1.48, 7.22; P = 0.003), while a HH from Uriri had a 2.7 times higher likelihood when compared to CK (OR = 2.69; 95% CI = 1.63, 44.44; p < 0.001). Male partner attendance at an antenatal care (ANC) visit was positively associated with U5 care utilization for fever (OR = 1.72; 95% CI = 0.56, 1.21; p = 0.007), while a history of health facility delivery and ability to identify warning signs were negatively associated with care seeking (facility delivery: OR = 0.50; 95% CI = 0.29, 0.86; p = 0.012; warning sign recognition: OR = 0.72; 95% CI = 0.49, 1.05; p = 0.090). For ARI, being from Uriri was positively associated with care utilization (OR = 2.13; 95% CI = 1.18, 3.83; p = 0.012). HHs in which the head of HH had a secondary education were 1.6 times more likely to seek care for a child´s respiratory illness (OR = 1.59; 95% CI = 1.03, 2.45; p = 0.037), and HHs visited by a CHW were 1.8 times more likely to seek care (OR = 1.84; 95% CI = 1.25, 2.70; p = 0.002). Larger HH size was negatively associated with care seeking for ARI (OR = 0.84, 95% CI = 0.74, 0.95, p = 0.006). In the case of diarrhea, the only variable positively associated with care seeking was being from a HH which had previously experienced the death of a child under five years (OR = 4.2, 95% CI = 1.1, 16.03, p = 0.036).

**Table 4:** logistic regression model for whether a head of household sought advice for their child at a health facility with fever, diarrhea, and respiratory illness: 2018/2019

Variable	Fever			Diarrhea			Respiratory illness		
	OR	95%CI	p-value	OR	95%CI	p-value	OR	95%CI	p-value
**Region**									
CK	Ref	--	--	Ref	--	--	Ref	--	--
NK	3.27	(1.48, 7.22)	0.003	0.95	(0.32, 2.83)	0.935	1.63	(0.78, 3.38)	0.190
EK	1.89	(1.16, 3.08)	0.011	1.02	(0.51, 1.99)	0.965	0.98	(0.57, 1.68)	0.959
SK	1.49	(0.93, 2.38)	0.095	0.99	(0.49, 1.98)	0.982	1.15	(0.67, 1.99)	0.603
Uriri	2.69	(1.63, 4.44)	<0.001	1.67	(0.74, 3.75)	0.218	2.13	(1.18, 3.83)	0.012
Age (per 10-year increase)	1.10	(0.86, 1.41)	0.431	1.07	(0.71, 1.59)	0.745	1.20	(0.89, 1.61)	0.229
**Marital status**									
Never married	Ref	--	--	Ref	--	--	Ref	--	--
Married monogamous	0.32	(0.08, 1.20)	0.091	0.52	(0.12, 2.30)	0.394	0.75	(0.26, 2.19)	0.609
Married polygamous	0.31	(0.07, 1.24)	0.097	0.43	(0.08, 2.09)	0.298	0.74	(0.23, 2.33)	0.609
*Other single	0.39	(0.09, 1.70)	0.214	0.28	(0.04, 1.71)	0.167	1.10	(0.30, 4.02)	0.885
**Education**									
Class 1 -8	Ref	--	--	Ref	--	--	Ref	--	--
Form 1-4	1.29	(0.87, 1.91)	0.199	1.02	(0.57, 1.84)	0.947	1.59	(1.03, 2.45)	0.037
Some college/post-graduate	1.90	(0.91, 3.95)	0.086	0.91	(0.36, 2.33)	0.848	1.30	(0.61, 2.76)	0.500
No education**	0.71	(0.13, 3.86)	0.691	--	--	--	0.18	(0.01, 2.56)	0.208
Household size (per 1-person increase)	0.93	(0.83, 1.05)	0.240	0.87	(0.73, 1.03)	0.112	0.84	(0.74, 0.95)	0.006
Household ever visited by CHW	1.13	(0.78, 1.63)	0.502	0.91	(0.54, 1.55)	0.735	1.84	(1.25, 2.70)	0.002
Last pregnancy was desired	1.20	(0.85, 1.69)	0.290	1.20	(0.72, 1.98)	0.477	1.35	(0.92, 1.98)	0.117
Child delivered at a health facility	0.50	(0.29, 0.86)	0.012	0.50	(0.22, 1.14)	0.102	0.66	(0.37, 1.18)	0.167
Attended 4 or more ANC visits	0.82	(0.56, 1.21)	0.320	1.46	(0.85, 2.50)	0.168	0.83	(0.55, 1.26)	0.394
Male partner ever attended ANC visit	1.72	(1.16, 2.55)	0.007	1.27	(0.72, 2.22)	0.408	1.25	(0.81, 1.92)	0.304
In last 5-yrs has had a child that died	1.18	(0.55, 2.48)	0.669	4.20	(1.1, 16.03)	0.036	1.53	(0.65, 3.58)	0.322
Current use of family planning	1.20	(0.78, 1.83)	0.399	0.85	(0.47, 1.57)	0.621	1.09	(0.68, 1.74)	0.724
Correctly recognizes ≥ 3 signs that indicate a child needs medical attention	0.72	(0.49, 1.05)	0.090	1.43	(0.85, 2.41)	0.173	0.97	(0.64, 1.45)	0.886

* Other single includes cohabitation, separated, divorced, and/or widowed; **the number of children with diarrhea whose head of household had no education was too small to be able to interpret

In logistic regression looking at prevalence of ARI alongside kitchen location and fuel type (Table 5), HHs with an in-house kitchen trended towards being more likely to have a child with ARI, compared to HHs with kitchens located outside the main house (OR = 1.23; 95% CI = 0.98, 1.52; p = 0.065). However, there was not a strong association with fuel type (OR = 1.2; 95% CI = 0.85, 1.69; p = 0.287).

**Table 5:** logistic regression for the association between a child having a respiratory illness and the location of their kitchen and cooking fuel type: 2018/2019

Variable	OR	95% CI	p-value
**Location of kitchen**			
Separate from main home	Ref	---	---
In-house kitchen	1.23	(0.98, 1.52)	0.065
**Cooking fuel**			
Non-smoke producing fuel	Ref	---	---
Firewood/charcoal	1.204	(0.85, 1.69)	0.287

## Discussion

Overall, the 2018/2019 data shows prevalence of fever, diarrhea, and ARI in children 0-59 months of age across the five sublocations surveyed in Migori County was 18%, 8%, and 14% respectively. This represents a sizable change in the reported prevalence of these conditions as compared to Nyanza Province as a whole in the 2014 KDHS, in which the prevalence of fever, diarrhea and ARI was 37.4%, 18.9%, and 9% respectively. We highlight further evidence here suggesting a decrease in the prevalence of these three conditions as seen in the data presented from NK over time, in which the prevalence of fever, diarrhea, and ARI decreased from 50%, 26% and 40% respectively in 2017 to 26%, 9%, and 24% respectively in 2018/2019.

In the decade prior to this latest survey, significant investments have been made by government as well as non-governmental organizations to address maternal, child and neonatal morbidity and mortality in the region. For example, in NK, Lwala began supporting the delivery of an integrated maternal, child, and newborn health service package in 2012, based on utilization of integrated community case management for childhood illness (iCCM) and the CHW AIM framework [9]. The intervention employed paid, trained, and supervised CHWs connected to primary health facilities that were recruited from existing government community health volunteer cadres. Additional emphasis was made to recruit and train local TBAs into the CHW pool, capitalizing on local knowledge and persons with a high level of trust and influence within the community. This intervention is characterized in other studies [11,15,21]. Over time, Lwala worked with CHWs to expand their services offered, including the addition of malaria community case management services, which were launched in the period between implementation of the 2017 and 2018/2019 surveys. Malaria community case management services included CHW-delivered malaria prevention information; fever education; testing for malaria using RDTs; malaria treatment; and referral to a health facility for children U5 suspected or confirmed as having malaria.

While causal relationships cannot be established through our series of cross-sectional household surveys, they do provide valuable insight into changes in health indices at a population-level in Migori County over time. For example, it is not surprising that having had a malaria RDT performed in children with fever in NK rose from 24% in 2017 to 88% in 2018/2019, corresponding to the period of dramatic expansion of the malaria community case management services in the intervention area. Furthermore, this expansion of malaria related services likely contributed positively to the overall decrease in reported fever prevalence in NK in 2018/2019 compared to 2017, as well as contributing to the more than three-fold higher likelihood of care seeking in NK for a child with fever compared to children surveyed from other areas in which the intervention had not yet been implemented at the time of the survey.

The decision or ability to use health services is multifaceted and influenced by societal norms and values, socioeconomic factors, and perceived need [22-25]. Understanding the factors which contribute to one´s healthcare seeking behaviors can provide useful information to increase uptake. We found that coverage of CHW visits to one´s home varied across our surveyed areas, with NK showing higher rates of CHW-visited HHs compared to the other surveyed regions. We also found that HHs visited by a CHW were nearly twice as likely to seek care at a health facility for their child with a respiratory illness. This finding is consistent with reports from elsewhere in which household visits by a CHW were associated with greater utilization of facility-based maternal and child health services [26-29]. Future interventions for preventing unnecessary childhood morbidity and mortality should consider employing professionalized CHW cadres.

We also found that febrile children were more likely to be taken to a health facility for a fever if their mother had been accompanied to ANC visits during her last pregnancy by her male partner. While there are many studies showing male participation in ANC visits to be a predictor of maternal care utilization [30-33]; this association between male ANC attendance and child care utilization is not well described and should be explored further in future studies. However, based on our findings we posit that just as initiatives designed to increase male involvement in ANC positively impact the woman´s behavior around seeking a facility delivery, these same initiatives very likely may translate into improved healthcare seeking behaviors for the U5 children living in the household. Future studies should explore this correlation and the impact it could have on U5 mortality.

The finding that a prior skilled delivery was associated with decreased care utilization for febrile children conflicts with findings in similar studies and is misaligned with our expectations that mothers who deliver in a facility would be more likely to seek care for children [22]. Increased ability to recognize three or more danger signs was inversely associated with care utilization for a child with fever in our study. Our findings conflict with a study at Kenyatta National Hospital in Nairobi in which increased knowledge of danger signs was associated with timely care seeking amongst patients [34]. However, in contrast, our findings are more in line with studies showing that fever education reduces clinic visits and increases caretaker confidence in home-based fever management [35,36]. We speculate that the recently initiated CHW-led malaria case management program, which includes fever education, may be playing a role in our findings, though this connection is not well understood.

The respondent´s education, specifically completion of secondary education, increased the likelihood of seeking care for respiratory illness by 1.59 times. This result is consistent with other studies showing caregiver education to be a predictor of healthcare utilization [22,24,37-40]. Our expectation is that education is associated with both higher socio-economic status as well as increased access to health information, increasing the ability to navigate the health system. Additionally, higher education rates for women are often associated with increased control over HH resources and may increase female caregivers´ ability to seek health services for their children.

The size of a HH is inversely related to care seeking for children with ARI. A similar study in Coastal Kenya found that individuals in a HH who were not members of the nuclear family were five times less likely to access medical care [41]. Together, this may highlight the specific vulnerability of children living in larger HHs. We recommend further study and an exploration of health programs that specifically target children in large HHs and programs that increase access to family planning services. For diarrhea, the only factor associated with increased care utilization was a recent death of a U5 child in the HH. A study in Kenya found that caretakers who knew a child who had died of bloody diarrhea were more likely to report diarrhea in the previous two weeks. Relatedly, caregivers who knew dehydration could lead to death, were more likely to seek care for their child [37]. We posit that this may suggest that caretakers who are better informed of the severe consequences of diarrhea and childhood illness are more inclined to identify diarrhea and seek treatment.

This study has several important limitations to consider. First, this study covers only one specific region in Kenya. While this specificity allows for granular understanding of the survey population, it also limits its generalizability. This study is cross-sectional in nature and does not allow for conclusions to be drawn about the causality of results nor does it allow us to examine health seeking behavior of the same HHs over time.

Despite these limitations, the study has several strengths. The study has a large sample size, allowing for a representative picture of the region. Additionally, the survey interviewers were local residents and were thus able to conduct the interview in the respondents´ preferred local language. Additionally, the study used standardized question sets which allowed for comparisons to the KDHS and other related studies in Kenya and beyond.

## Conclusion

This study builds on a body of evidence demonstrating the potential for well-designed CHW programs to improve access and utilization of primary health services. We found that HHs visited by CHWs were nearly two times more likely to seek care for ARI, with HHs in the intervention area experiencing higher rates of CHW visits than other regions. Following the implementation of a CHW-led malaria program, RDT usage increased from 24% to 88% amongst children with fever, while fever prevalence decreased. Further, HHs in the intervention area were three times more likely to seek care for fever. Given these findings, we encourage local policy makers to further align community health interventions with the CHW AIM tool and WHO guidelines. Additionally, we recommend further study of the incorporation of traditional birth attendants within professionalized CHW cadres. Overall, the predictors of care utilization varied by symptom set. As such, we suggest that programs seeking to increase under-five care utilization should be contextualized by the regional burden of disease.

### What is known about this topic

- CHW visits are associated with increased care utilization across a range of maternal and child health services;
- Male partner attendance of ANC visits is a predicator of maternal health care utilization and facility-based delivery;
- Caregiver education is a predicator of healthcare utilization amongst children and households.

### What this study adds

- The study provides hyperlocal data for the population in Migori, Kenya, providing specific insights for national and subnational policy makers and implementers;
- The study found varied results by disease burden, suggesting a need for targeted approaches to under-five care utilization;
- The study builds upon literature of CHW home visits, caregiver education, male attendance of ANC visits, and other common variables as predictors of care utilization and associates these with care-seeking for specific conditions amongst children under five.

## Annexes

**Annex 1**: demographic, population-based household assessment of livelihoods and health among communities in Rongo Sub-County, Migori County, Kenya over multiple time points (2018-2022) - questions: staff_ID - HSHLD_Q14 (PDF-96kb)

**Annex 2**: demographic, population-based household assessment of livelihoods and health among communities in Rongo Sub-County, Migori County, Kenya over multiple time points (2018-2022) - questions: HSHLD_Q152 - CHH_Q14 (PDF-97kb)

**Annex 3**: demographic, population-based household assessment of livelihoods and health among communities in Rongo Sub-County, Migori County, Kenya over multiple time points (2018-2022) - questions: CHH_Q14 - end of surveys (PDF-102kb)

## References

[1] World Health Organization (2019). Child mortality and causes of death.

[2] Wang H, Bhutta ZA, Coates MM, Coggeshall M, Dandona L, Diallo K (2016). Global, regional, national, and selected subnational levels of stillbirths, neonatal, infant, and under-5 mortality, 1980-2015: a systematic analysis for the global burden of disease study 2015. Lancet.

[3] Macharia PM, Giorgi E, Thuranira PN, Joseph NK, Sartorius B, Snow RW (2019). Sub national variation and inequalities in under-five mortality in Kenya since 1965. BMC Public Health.

[4] (2015). Kenya National Bureau of Statistics Ministry of Health/Kenya, National AIDS Control Council/Kenya, Kenya Medical Research Institute, National Council for Population and Development/Kenya, and ICF International. Kenya demographic and health survey 2014.

[5] United Nations Take action for the sustainable development goals.

[6] Keats EC, Macharia W, Singh NS, Akseer N, Ravishankar N, Ngugi AK (2018). Accelerating Kenya´s progress to 2030: understanding the determinants of under-five mortality from 1990 to 2015. BMJ Glob Health.

[7] Government of Kenya Big four agenda implementation status report 2018/19.

[8] World Health Organization (2018). WHO guideline on health policy and system support to optimize community health worker programmes.

[9] Ballard M, Bonds MH, Burey J, Dini HSF, Foth J, Furth R (2018). CHW AIM: updated program functionality matrix for optimizing community health programs.

[10] World Health Organization Community health worker assessment and improvement matrix (CHW AIM): a toolkit for improving community health worker programs and services.

[11] Starnes JR, Wamae J, Okoth V, Ressler DJ, Were V, Were LPO (2021). Population-based socio-demographic household assessment of livelihoods and health among communities in Migori County, Kenya over multiple timepoints (2021, 2024, 2027): a study protocol. Plos One.

[12] Starnes JR, Gravio CD, Irlmeier R, Moore R, Okoth V, Rogers A (2021). Characterizing multidimensional poverty in Migori County, Kenya and its association with depression. Plos One.

[13] Starnes JR, Chamberlain L, Sutermaster S, Owuor M, Okoth V, Edman W (2018). Under-five mortality in the Rongo Sub-County of Migori County, Kenya: experience of the Lwala Community alliance 2007-2017 with evidence from a cross-sectional survey. Plos One.

[14] Morris M, Okoth V, Prigmore HL, Ressler DJ, Mbeya J, Rogers A (2020). The prevalence of interpersonal violence (IPV) against women and its associated variables: an exploratory study in the Rongo Sub-County of Migori County, Kenya. J Interpers Violence.

[15] Moon TD, Okoth V, Starnes JR, Opiyo E, Ressler DJ, Mbeya J (2021). Determinants of modern contraceptive prevalence and unplanned pregnancies in Migori County, Kenya: results of a cross-sectional household survey. African Journal of Reproductive Health.

[16] Harris DR, Lemeshow S (1991). Evaluation of the EPI survey methodology for estimating relative risk. World Health Stat Q.

[17] Bostoen K, Chalabi Z (2006). Optimization of household survey sampling without sample frames. Int J Epidemiol.

[18] Grais RF, Rose AM, Guthmann J-P (2007). Don´t spin the pen: two alternative methods for second-stage sampling in urban cluster surveys. Emerg Themes Epidemiol.

[19] Harris PA, Taylor R, Thielke R, Payne J, Gonzalez N, Conde JG (2009). Research electronic data capture (REDCap)--a metadata-driven methodology and workflow process for providing translational research informatics support. J Biomed Inform.

[20] Harris PA, Taylor R, Minor BL, Elliott V, Fernandez M, O´Neal L (2019). The REDCap consortium: building an international community of software platform partners. J Biomed Inform.

[21] Heerboth SA, Hennessey C, Omondi B, Wafula M, Mbeya J, Rogers A (2020). Knowledge of obstetric and neonatal danger signs among community health workers in the Rongo Sub-County of Migori County, Kenya: results of a community-based cross-sectional survey. Afr J Reprod Health.

[22] Bayham M, Blevins M, Lopez M, Olupona O, González-Calvo L, Ndatimana E (2017). Predictors of health-care utilization among children 6-59 months of age in Zambézia Province, Mozambique. Am J Trop Med Hyg.

[23] Marmot M (2005). Social determinants of health inequalities. Lancet.

[24] Osaki K, Kosen S, Indriasih E, Pritasari K, Hattori T (2015). Factors affecting the utilisation of maternal, newborn, and child health services in Indonesia: the role of the maternal and child health handbook. Public Health.

[25] Tinuade O, Iyabo R-A, Durotoye O (2010). Health-care-seeking behaviour for childhood illnesses in a resource-poor setting. J Paediatr Child Health.

[26] Edmond KM, Yousufi K, Anwari Z, Sadat SM, Staniczai SM, Higgins-Steele A (2018). Can community health worker home visiting improve care-seeking and maternal and newborn care practices in fragile states such as Afghanistan: a population-based intervention study. BMC Med.

[27] Geldsetzer P, Mboggo E, Larson E, Lema IA, Magesa L, Machumi L (2019). Community health workers to improve uptake of maternal healthcare services: a cluster-randomized pragmatic trial in Dar es Salaam, Tanzania. PLoS Med.

[28] Blanchard AK, Prost A, Houweling TAJ (2019). Effects of community health worker interventions on socioeconomic inequities in maternal and newborn health in low-income and middle-income countries: a mixed-methods systematic review. BMJ Glob Health.

[29] Stansert Katzen L, Tomlinson M, Christodoulou J, Laurenzi C, le Roux I, Baker V (2020). Home visits by community health workers in rural South Africa have a limited, but important impact on maternal and child health in the first two years of life. BMC Health Serv Res.

[30] Mohammed BH, Johnston JM, Vackova D, Hassen SM, Yi H (2019). The role of male partner in utilization of maternal health care services in Ethiopia: a community-based couple study. BMC Pregnancy Childbirth.

[31] Aguiar C, Jennings L (2015). Impact of male partner antenatal accompaniment on perinatal health outcomes in developing countries: a systematic literature review. Matern Child Health J.

[32] Yargawa J, Leonardi-Bee J (2015). Male involvement and maternal health outcomes: systematic review and meta-analysis. J Epidemiol Community Health.

[33] Mullany BC, Becker S, Hindin M (2007). The impact of including husbands in antenatal health education services on maternal health practices in urban Nepal: results from a randomized controlled trial. Health Educ Res.

[34] Wambui WM, Kimani S, Odhiambo E (2018). Determinants of health seeking behavior among caregivers of infants admitted with acute childhood illnesses at Kenyatta National Hospital, Nairobi, Kenya. Int J Pediatr.

[35] Sarrell M, Cohen HA, Kahan E (2002). Physicians´, nurses´, and parents´ attitudes to and knowledge about fever in early childhood. Patient Educ Couns.

[36] Robinson JS, Schwartz M-LM, Magwene KS, Krengel SA, Tamburello D (1989). The impact of fever health education on clinic utilization. Am J Dis Child.

[37] Omore R, O´Reilly CE, Williamson J, Moke F, Were V, Farag TH (2013). Health care-seeking behavior during childhood diarrheal illness: results of health care utilization and attitudes surveys of caretakers in Western Kenya, 2007-2010. Am J Trop Med Hyg.

[38] Buor D (2003). Mothers´ education and childhood mortality in Ghana. Health Policy.

[39] Caldwell JC (1994). How is greater maternal education translated into lower child mortality. Health Transition Review.

[40] Chakrabarti A (2012). Determinants of child morbidity and factors governing utilization of child health care: evidence from rural India. Applied Economics.

[41] Ngugi AK, Agoi F, Mahoney MR, Lakhani A, Mang´ong´o D, Nderitu E (2017). Utilization of health services in a resource-limited rural area in Kenya: prevalence and associated household-level factors. PLoS One.

